# Analysis of laboratory adhesion studies in eroded enamel and dentin: a scoping review

**DOI:** 10.1080/26415275.2021.1884558

**Published:** 2021-02-15

**Authors:** Madalena Belmar da Costa, António H. S. Delgado, Teresa Pinheiro de Melo, Tomás Amorim, Ana Mano Azul

**Affiliations:** a Instituto Universitário Egas Moniz (IUEM), Almada, Portugal; b Division of Biomaterials and Tissue Engineering, UCL Eastman Dental Institute, London, UK; c Centro de Investigação Interdisciplinar Egas Moniz (CiiEM), Almada, Portugal

**Keywords:** Adhesion, bonding, bond strength, dentin, dental erosion, enamel

## Abstract

**Aim:**

To summarize and report laboratory studies of adhesion in eroded substrates, which used bond strength as an outcome measure. To determine the strategies available to overcome bonding difficulties, the quality and consistency of the methodology and to find evidence gaps.

**Materials and Methods:**

The present review followed PRISMA-ScR guidelines. A search was conducted on PubMed/Medline, Scopus and EMBASE (Ovid) databases to identify published peer-reviewed papers (2010–2020). For final qualitative synthesis, 29 articles were selected which respected the inclusion criteria. Data charting was carried out, independently, by two reviewers and quality assessment of the articles was performed.

**Results:**

The primary studies included fall into four major categories: comparison of restorative materials and application modes, enzymatic inhibitors, surface pretreatments or remineralization strategies. Most studies found evaluated dentin (76%), while 17% evaluated enamel, and 7% evaluated both substrates. The majority of the studies reported an effective intervention (83%). Bond strength to eroded dentin is significantly reduced, while in enamel erosion is beneficial. The bond strength to eroded dentin is material-dependent and favored in systems containing 10-MDP. Great disparities among the erosion models used were found, with citric acid in different concentrations being the preferred method, although standardization is lacking.

**Conclusions:**

Adhesives containing 10-MDP show beneficial results in eroded dentin, and surface preparation methods should be considered. Studies which evaluated adhesion to eroded enamel/dentin show high heterogeneity in what concerns aims and methodology. Strategies that focus on remineralizing dentin and strategies to protect bond longevity in this substrate require further research.

## 1. Introduction

Erosion in enamel and dentin is considered an increasingly complex challenge in dentistry [1]. According to Bartlett, Okunseri and Lussi, the prevalence of dental erosion is high and is present in approximately 30% of the world population. Dental erosion is also more common among men [2–5].

The success of operative dentistry is largely determined by the correct understanding of the chemical and biological processes that govern the tooth structure. Only this way is possible to understand pathological changes in the oral cavity and, consequently, adapt clinical procedures to the case [6].

Enamel and dentin are highly mineralized tissues, made up of an organized inorganic matrix of hydroxyapatite crystals [7]. Enamel comprises 96 wt% of hydroxyapatite crystals, while the remaining 4% are water and residual organic content [7–9]. Alike enamel, there is also an inorganic matrix in dentin, although in lesser quantity, surrounding and protecting the organic content. This is mostly type-I collagen, responsible for making dentin a challenging substrate to bond to [10]. Despite their apparent similarities, they each have different coping mechanisms and regeneration potentials, in response to the various aggressions they may be subjected to in the oral environment [8]. These include trauma, caries, abrasion, attrition and erosion [11].

Erosion is described in the literature as a noncarious progressive lesion linked to the dissolution of hard tissues by acids which are not bacterial by-products [12]. This gradual dissolution leads to the weakening of the enamel and increased susceptibility to abrasion or attrition, yet it remains remineralizable [13]. However, a prolonged exposure to acids may render the enamel unable to regenerate, leaving it permanently affected, ultimately impacting on the underlying dentin [14]. Thus, and taking into account the prevalence of this phenomenon, it is important to adopt rehabilitation strategies in order to guarantee the protection of the dental hard tissues [15]. Erosive defects may even elicit pain, in certain clinical scenarios, where dentin is severely affected, requiring immediate intervention [1].

In light of the current available evidence, the existing strategies rely on adhesive protocols and novel biomimetic approaches which benefit from advances in nanotechnology. These include new bioactive polymers, fillers or toothpastes which aid calcium-phosphate remineralization [6,16–20].

Bonding to an eroded substrate and its predictability will vary depending on whether it is enamel or dentin. Due to aforementioned factors, they behave differently during the adhesive process [21,22]. In enamel, the erosive process seems to be beneficial for adhesion since it promotes the creation of micro and macroporosities that facilitate resin penetration and retention, in a high surface energy substrate [13,17,21]. In contrast, there is increased difficulty in bonding to eroded dentin [21,23,24]. Occurrences such as a hypermineralization layer and tubular occlusion, lead to a weak reactionary ability of this substrate. Due to this, resin impregnation is impaired, ultimately leading to a compromise in the bonding procedure [11,25–27]. Consensus is yet to be reached regarding the best materials or strategies available to improve bond strength to eroded substrates. Furthermore, optimization of such bonding strategies contribute to durable restorations, as questions may arise regarding longevity of bonded eroded substrates [23,26]. Such interventions are pivotal in severe erosion cases, which involve deep dentin, as this disfavors the long-term prognosis of the restoration.

Therefore, taking into account that dental erosion reflects one of the greatest challenges today in oral rehabilitation [1,28], the objective of this review is to sum and report laboratory studies of adhesion in eroded substrates, in order to understand what has been done, to summarize the knowledge in the field, assess the quality of the studies performed and to identify gaps in the evidence. This will inform and direct future research.

## 2. Materials and methods

### 2.1. Search strategy

This scoping review was conducted according to the PRISMA-Scr Statement criteria (Preferred Reporting Items for Scoping Reviews) [29]. To identify the primary review question, the PCC framework of the Joanna Briggs Institute was adopted, where P (Population) was defined as restorations in enamel/dentin, C (Concept) was defined as bonding to eroded enamel/dentin and C (Context) were laboratory studies [30]. A search strategy was developed for OVID (EMBASE), Medline/PubMed and Scopus databases, with keywords obtained from Medical Subject Headings (MeSH) and additional free keywords. These were combined with Boolean operators as follows: ((Dental erosion) OR (Tooth erosion)) AND (Adhes* OR Bond* OR Materials testing OR Tensile strength OR Dental bonding* OR Dentin-Bonding Agents* OR resin-dentin). The electronic search covered peer-reviewed papers that were published in the last 10 years (2010–2020), as ideas in adhesive dentistry are rapidly abandoned, the most relevant research will be the latest. There was no language restriction. The last search was conducted on 16 September 2020. Records were retrieved and potentially relevant titles and abstracts were selected, followed by full-text reading and inclusion.

### 2.2. Eligibility criteria

Only laboratory studies that tested bonding of a dental material to eroded enamel or dentin were considered for this review. To respect the inclusion criteria, the bonding procedure had to be carried out in an already eroded substrate. Substrates that suffered erosion protocols after bonding were excluded, as this is not the aim. Studies were only included if their outcome measured any setup of bond strength test (tensile, shear or push-out), which is a gold-standard measure of dental adhesive longevity. The focus were studies of eroded substrates for restorative purposes, using resin composite. Studies which focused on bond strength of orthodontic brackets were excluded. Only studies that used human permanent teeth or bovine teeth were considered eligible. Clinical studies or other animal studies were excluded.

### 2.3. Study selection and data processing

The data were retrieved from the databases and organized using Mendeley Desktop software (v.1.19.4), where duplicates were removed. Screening was done by three reviewers, in triplicate, and disagreements were resolved by consensus. Data charting was developed by two reviewers (M.B.C. and A.D), which was then independently charted. All relevant information was extrapolated to a Microsoft Excel spread sheet (v. 16.37, Microsoft, USA) which included: author and date of study, country, substrate used, sample size, materials, intervention, test and conclusion. These are summarized in Table 1. Reasons for exclusion of studies following full-text reading were recorded.

**Table 1.** Laboratory adhesion studies in eroded substrates included in the scoping review.

Author	Country	Substrate	Sample size	Adhesive	Intervention	Test	Statistical Significance*	Conclusion
Siqueira et al. [32]	Brazil	Dentin	*n* = 7	Prime&Bond Elect Scotchbond U AdheSE U	Use of primers containing MMP inhibitors RFV and PAA	µTBS (24h and 24 months) Nanoleakage Nanohardness	Significant	The inclusion of cross-linking agents contributed to improving the immediate properties and stabilized the adhesive interface long-term.
Ferreti et al. [33]	Brazil	Dentin	*n* = 10	Clearfil SE	Cigarette smoke exposure and erosive protocol on bond strength and nanohardness	µSBS (24h) SEM Microhardness	Not significant	The erosive protocol studied did not alter the bond strength of the self-etch, when compared to sound dentin.
Krithi et al. [34]	India	Dentin	*n* = 15	Adper SB 2 Clearfil SE	Effect of remineralization of NaF, CPP-AP and NovaMin	µSBS (24h)	Significant	The type of adhesive influences bond strength to eroded dentin. Sodium fluoride and NovaMin showed improvement in bond strength results.
Murase et al. [35]	Japan	Enamel Dentin	*n* = 10	Fusio Liquid Dentin LLB-CR6 Clearfil SE Bond	Test a new bond strength setup and self-adhesive composites to eroded enamel/dentin	TBS (24h) Microhardness	Significant	Self-adhesive flowable composites show no differences between eroded and non-eroded substrates and may be a *via*ble strategy clinically.
Costa et al. [36]	Brazil	Dentin	*n* = 6	Clearfil SE	Pretreatment with MMP inhibitors epigallocatechin-3-gallate and 2% CHX	µTBS (24h and 6 months)	Significant	The use of 2% chlorhexidine digluconate negatively affected eroded and non-eroded substrates, while epigallocatechin showed no difference.
Yabuki et al. [37]	Japan	Enamel	*n* = 10	AdheSE U All Bond U Scotchbond U	Different universal adhesives in eroded enamel	SBS (24h) CLSM	Significant	Bond strength to eroded enamel, in all adhesives, is higher than to sound enamel.
Zumstein et al. [38]	Switzerland	Dentin	*n* = 23	Clearfil SE Scotchbond U	Pretreatment with SnCl2/AmF	µTBS (24h and 12 months)	Significant	Bonding to eroded dentin is significantly lower than to sound. The pretreatment was not signifcant. No differences among adhesives, but aging was significantly lower.
Siqueira et al. [39]	Brazil	Dentin	*n* = 8	Adper SB 2 Scotchbond U	Effect of deproteinization (5.2% NaOCl)	µTBS (24h and 36 months) Nanoleakage	Significant	Deproteinization influences the stability of a resin-eroded dentin interface in the tested adhesives.
Moda et al. [40]	Brazil	Dentin	*n* = 9	RelyX U200	Effect of 3 surface treatments: EDTA, 20% polyacrylic acid and 2% CHX	µTBS (24h and 8 months)	Significant	In general, the surface pretreatments promoted an increase in bond strength to the eroded substrate, immediate and long-term.
Augusto et al. [41]	Brazil	Dentin	*n* = 10	FuturaBond M+	The effect of different application modes of a universal adhesive to eroded, deproteinized and abraded dentin	µTBS (24h and 6 months)	Significant	NaOCl at 10% as a deproteinizing agent increased the bond strength results and maintained stability to eroded dentin. Differences were found regarding the application mode.
Siqueira et al. [42]	Brazil	Dentin	*n* = 5	AdheSE U AllBond Universal Ambar Universal Clearfil Universal Futurabond U One Coat 7 U Peak Universal B Prime & Bond Elect Scotchbond U Xeno Select	Effect of different universal adhesives on eroded dentin	µTBS (24h)	Significant	MDP-containing adhesives showed better results in both application modes in sound and eroded substrates.
Deari et al. [43]	Switzerland	Dentin	*n* = 6	Optibond FL	Effect of different pretreatments: bur abrasion, 10% NaOCl, 2% CHX and prolonged application of the primer (60 s)	µTBS (24h)	Significant	Bur abrasion and pretreatment with NaOCl achieved higher bond strengths to eroded dentin.
Forgerini et al. [44]	Brazil	Dentin	*n* = 10	Adper SB Plus Clearfil SE Scotchbond U	Immediate and long-term performance of a universal adhesive	µSBS (24h and 6 months)	Significant	The performance is irrespective of application mode, however it is less effective in eroded dentin.
Flury et al. [45]	Switzerland	Dentin	*n* = 16	Scotchbond 1 XT Optibond FL	Artificially eroded samples with or without 1% BAC incorporation	SBS (24h and 12 months) SEM	Significant	BAC increased the 24 h SBS to eroded dentin and had no effect on the 12 month results.
Frattes et al. [46]	Brazil	Enamel Dentin	*n* = 22	Scotchbond U	Erosive conditions and acid-etching	µTBS (24h) SEM	Significant	Erosive challenge increased µTBS for the adhesive to enamel but not to dentin.
Giacomini et al. [47]	Brazil and USA	Dentin	*n* = 90	Adper SB Universal	Erosive conditions, acid etching and pretreatment with 2% CHX	µTBS (24h)	Significant	All tested factors were significant. Lower µTBS in artificial carious dentin and eroded dentin.
Bergamin et al. [48]	Brazil	Dentin	*n* = 10	Adper SB 2 Clearfil SE	Pretreatment with an arginine-containing toothpaste followed by different adhesives	µTBS (24h)	Not significant	No differences were found between toothpastes, adhesives or interaction between both in eroded dentin.
Maeda et al. [49]	Brazil	Dentin	*n* = 10	Adper SB 2 Adper SE Plus	Nd:YAG laser irradiation and its effect on bond strength to eroded and non-eroded substrate	SBS (24h)	Significant	Under erosive challenge, Nd:YAG laser seems to be beneficial in improving bond strength. An etch-and-rinse approach is preferred.
Giacomini et al. [50]	Brazil	Enamel	*n* = 8	Adper Scotchbond Adper SB 2 Clearfil SE	Effect of different adhesives on eroded and abraded enamel	µTBS (24h)	Significant	Bond strength to eroded enamel is not affected, although it is material-dependent. Authors state phosphoric acid suffices in this substrate.
Francisconi-dos-Rios et al. [51]	Brazil	Dentin	*n* = 7	Adper SB 2	Pretreatment with 2% CHX solution on bond strength to eroded dentin compared to sound dentin	µTBS (24h and 6 months) CLSM	Significant	Eroded dentin had lower µTBS. 2% CHX on bond strength conservation to both eroded and sound dentin was not effective except after 6 months aging.
Cruz et al. [52]	Brazil	Dentin	*n* = 10	Adper Easy One Adper SB 2 Clearfil SE	Effect of different adhesives and different time points on eroded and sound dentin	µSBS (24h and 6 months)	Not significant	No differences in bonding performance of the adhesives tested.
Machado et al. [53]	Brazil	Dentin	*n* = 10	Adper SB 2	Pretreatment with 2% CHX as an MMP inhibitor	µSBS (1 and 6 months)	Significant	2% CHX was not effective neither on sound nor eroded dentin at 1 and 6 months.
Wang et al. [13]	Brazil	Enamel	*n* = 13	Adper SB 2	Simulating abrasion with toothbrushing, erosion and erosion + abrasion simultaneously	µTBS (24h)	Not significant	Neither erosive nor abrasive lesions resulting from the *in situ* challenges affected the resin-enamel bonding.
Casas-Apayco et al. [54]	Brazil	Enamel	*n* = 8	Adper SB 2	Effect of different cola drinks on the adhesion to eroded enamel	µTBS (24h)	Significant	All cola drinks reduced the bond strength to enamel.
Flury et al. [55]	Switzerland	Dentin	*n* = 20	Clearfil SE	Pretreatment with NaF or SnF on eroded dentin	µTBS (24h) and SEM	Significant	Treatment of erosively demineralized dentin with a NaF solution or SnF mouth rinse increased bond strength of resin composite.
Lenzi et al. [56]	Netherlands	Enamel	*n* = 12	Adper SB 2 Vitro fil LC	Comparison of an etch-and-rinse adhesive to a resin-modified glass ionomer cement in eroded enamel	SBS (24h)	Significant	When bonding to eroded enamel, bond strength of the etch-and-rinse increases while the glass ionomer shows no difference.
Ramos et al. [57]	Brazil and Germany	Dentin	*n* = 104	Clearfil SE Scotchbond U	Pretreatment with Er,Cr:YSGG laser irradiation	µTBS (24h) and SEM	Significant	The surface treatment with Er,Cr:YSGG laser prior to bonding with a self-etching adhesive system improves bond strength to eroded dentin.
Zimmerli et al. [58]	Switzerland	Dentin	*n* = 20	Clearfil SE Optibond FL	Surface preparation techniques on eroded dentin	µTBS (24h and 12 months) and SEM	Significant	Erosion reduced bond strength in all groups but this effect was less prominent when eroded dentin was prepared by diamond bur.
Cruz et al. [59]	Brazil	Dentin	*n* = 6	Adper SB 2 Ketac Molar Easy Mix Vitremer	Comparison of an etch-and-rinse to glass ionomer cements in eroded dentin	µSBS (24h)	Not significant	All materials behaved equally when comparing eroded to non-eroded substrates.

*Significance was considered if the null hypothesis of the study was rejected by statistical analysis. µSBS: microshear bond strength; µTBS: Microtensile bond strength; AmF: amide fluoride; BAC: benzalkonium chloride; CHX: chlorhexidine; CLSM: Confocal Laser Scanning Microscopy; CPP-AP: casein phosphopeptide-amorphous calcium phosphate nanocomplex; EDTA : ethylenediaminetetraacetic acid; Er,Cr:YSGG: erbium, chromium: yattrium-scandium-gallium-garnet laser; LLB-CR6: experimental adhesive; NaOCl: sodium hypochlorite; NaF: sodium fluoride Nd:YaG: neodymium-doped yttrium aluminium garnet; MMP: matrix metalloproteinases; PAA: Proanthrocyanidins; RFV: riboflavin; SBS: shear bond strength; SEM: Scanning electron microscopy; SnF: strontium fluoride; TBS: tensile bond strength.

### 2.4. Quality assessment – risk of bias

Although risk of bias assessment is optional, according to the PRISMA extension for Scoping Reviews, it can be performed depending on the nature of the review question [29]. In this case, a descriptive analysis of methodological quality in the laboratory research conducted in the primary studies is relevant. Thus, to assess this, in order to guide future research, seven parameters were chosen to be analysed in a Yes/No scale, similarly to Montagner et al. [31]. When insufficient information was provided, the item was classified with a ‘No’. These were: sample randomization, sample size calculation, use of sound teeth, the presence of a control group, reproducibility of the erosion protocol, use of materials according to the instructions and blinding of the operator on the test machine. Studies that scored at least three parameters with ‘No’ were classified as moderate risk, while more than three parameters were classified as having high risk of bias due to methodological flaws or uncertainty. Plots were built using the RoBvis 2.0 visualization tool (https://mcguinlu.shinyapps.io/robvis/).

## 3. Results

### 3.1. Study inclusion

**Figure 1.**
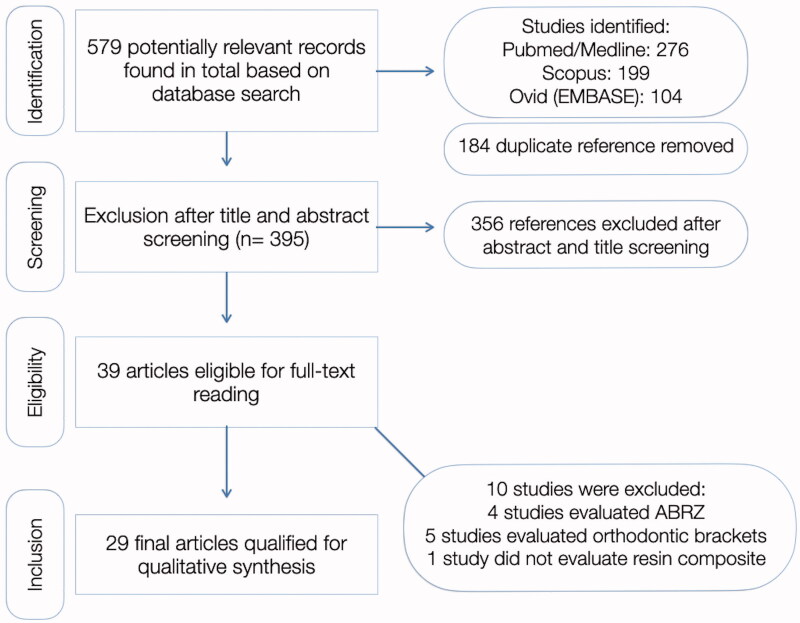
PRISMA-ScR flowchart used for the scoping review. Reasons for study exclusion included: studies which looked at acid-base resistance zones (ABRZ), evaluating erosion after the restorative procedure, studies which evaluated adhesion to orthodontic brackets, or studies which did not use resin composite as a restorative material.

In total, 579 articles were identified in all databases. Out of these, 184 were duplicates and were subsequently removed. A total of 39 records remained after title and abstract screening, out of which 10 were excluded for reasons mentioned in Figure 1, which illustrates the flow diagram of study selection. All primary studies included and data recorded for each are shown in Table 1. One study was also excluded from this analysis because the author could not be contacted and the full-text could not be retrieved.

### 3.2. Study characteristics

Most studies that investigated adhesion in eroded substrates focused on dentin (22/29 − 76%). Only five studies used enamel as a substrate (17%), and only two evaluated both (7%). As a bond strength testing setup, microtensile bond strength was the preferred choice (18/29 − 62%), followed by microshear (6/29 − 21%), shear (4/29 − 14%), and one study used a macrotensile setup (3%). For studies in which long-term bond strength was studied in addition to immediate bond strength (24 h), storage in water was the preferred method (8/12 − 66%). This was followed by artificial saliva (3/12 − 25%), and one study used 5000 cycles of thermocycling.

### 3.3. Materials and interventions

**Figure 2.**
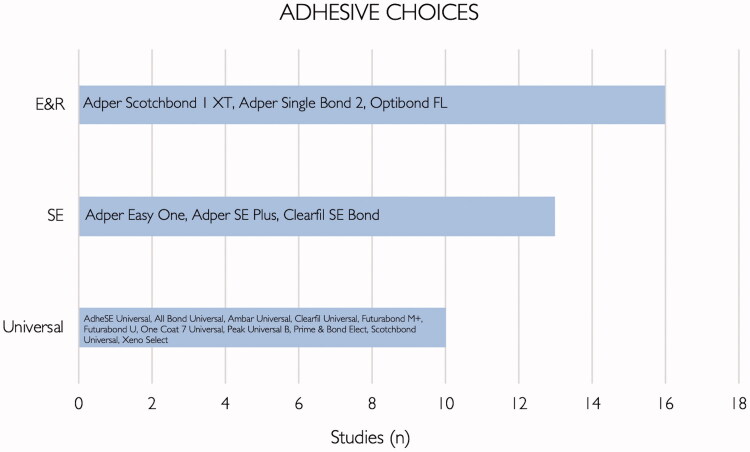
List of adhesives and their respective categories included in the primary studies.

Regarding the choice of adhesives in the primary studies that were identified, most authors used etch-and-rinse adhesives (16/29 − 55%), followed by self-etch adhesives (13/29 − 45%) and finally universal adhesives (10/29 − 34%) – Figure 2. Two studies evaluated glass ionomer cements (7%), and one study evaluated a luting cement (3%). The interventions investigated in primary studies included: testing of matrix metalloproteinases (MMP) inhibitor strategies, comparison of restorative materials and application modes, remineralization strategies and surface pretreatments.

### 3.4. Erosion protocols

The different erosion protocols used in the primary studies and respective frequencies can be seen in Table 2. The citric acid used in the 16 studies that were included varied between pH 2.1-3.75.

**Table 2.** Erosion protocols discriminated by study and frequency of appearance (n).

Solution used for erosion	Studies (n)	References
Citric acid pH range [2.1–3.75]	16	Siqueira et al. [32], Ferreti et al. [33], Krithi et al. [34], Costa et al. [36], Yabuki et al. [37], Zumstein et al. [38], Siqueira et al. [39], Augusto et al. [41], Siqueira et al. [42], Deari et al. [43], Flury et al. [45], Frattes et al. [46], Bergamin et al. [48], Flury et al. [55], Ramos et al. [57], Zimmerli et al. [58]
Coca-Cola, Coca-Cola Light or Coca-Cola Zero	10	Siqueira et al. [32], Murase et al. [35], Siqueira et al. [42], Forgerini et al. [44], Francisconi-dos-Rios et al. [51], Machado et al. [53], Wang et al. [13], Casas-Apayco et al. [54], Lenzi et al. [56], Cruz et al. [59]
Sprite **Zero, Sprite** Light	2	Maeda et al. [49], Cruz et al. [52]
Orange Juice	2	Giacomini et al. [47], Giacomini et al. [50]
HCl-pepsin (Hydrochloric acid and pepsin)	1	Moda et al. [40]

### 3.5. Quality assessment – risk of bias

**Figure 3.**
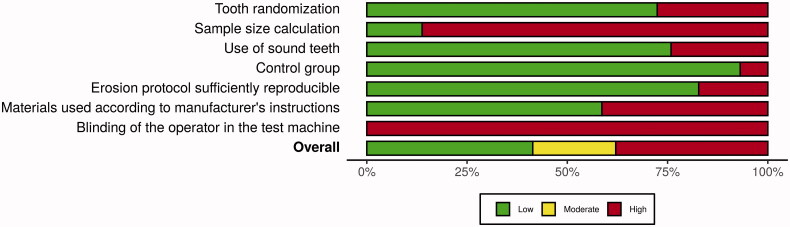
Weighted summary plot of the seven domains included in the risk of bias assessment.

The output of the quality assessment showing the results for each parameter that was evaluated is shown in Table 3. Twelve studies were classified as having low risk of bias (12/29 − 41%), and six studies were classified as moderate risk (6/29 − 21%). The remaining 11 studies were classified as having high risk of bias (11/29 − 38%). Only Yabuki et al. [37], Augusto et al. [41], Siqueira et al. [42] and Flury et al. [55] reported sample size calculation. The last parameter evaluated, blinding of the operator in the test machine, was not performed on any of the studies evaluated in this review. The weighted summary plot of each parameter can be seen in Figure 3.

**Table 3.** Quality assessment of the primary studies included in this review, using a Yes/No scale for seven different domains.

Study	Sample randomization	Sound teeth	Sample size calculation	Control group	Erosion protocol reproducible	Adhesives according to manufacturers instructions	Blinding of the operator in the test machine	Overall
Siqueira et al. [32]	Y	Y	N	N	Y	Y	N	Moderate
Ferreti et al. [33]	N	N	N	Y	Y	Y	N	High
Krithi et al. [34]	Y	N	N	Y	Y	Y	N	Moderate
Murase et al. [35]	N	N	N	Y	Y	N	N	High
Costa et al. [36]	Y	Y	N	Y	Y	Y	N	Low
Yabuki et al. [37]	N	N	Y	Y	N	N	N	High
Zumstein et al. [38]	N	Y	N	Y	Y	N	N	High
Siqueira et al. [39]	Y	Y	N	Y	Y	N	N	Moderate
Moda et al. [40]	N	N	N	Y	Y	N	N	High
Augusto et al. [41]	Y	Y	Y	Y	Y	Y	N	Low
Siqueira et al. [42]	Y	Y	Y	Y	Y	Y	N	Low
Deari et al. [43]	Y	Y	N	Y	Y	Y	N	Low
Forgerini et al. [44]	Y	Y	N	Y	Y	Y	N	Low
Flury et al. [45]	N	Y	N	Y	N	N	N	High
Frattes et al. [46]	Y	Y	N	Y	Y	Y	N	Low
Giacomini et al. [47]	Y	Y	N	Y	Y	Y	N	Low
Bergamin et al. [48]	Y	Y	N	Y	Y	Y	N	Low
Maeda et al. [49]	Y	Y	N	Y	N	Y	N	Moderate
Giacomini et al. [50]	Y	Y	N	Y	Y	N	N	Moderate
Francisconi-dos-Rios et al. [51]	N	Y	N	Y	Y	N	N	High
Cruz et al. [52]	Y	Y	N	Y	Y	Y	N	Low
Machado et al. [53]	Y	Y	N	Y	N	N	N	High
Wang et al. [13]	Y	Y	N	Y	Y	N	N	Moderate
Casas-Apayco et al. [54]	Y	Y	N	Y	N	N	N	High
Flury et al. [55]	Y	Y	Y	Y	Y	Y	N	Low
Lenzi et al. [56]	Y	Y	N	Y	Y	Y	N	Low
Ramos et al. [57]	Y	N	N	N	Y	N	N	High
Zimmerli et al. [58]	N	N	N	Y	Y	Y	N	High
Cruz et al. [59]	Y	Y	N	Y	Y	Y	N	Low

## 4. Discussion

The number of patients that show signs of erosion has been increasing and therefore, it is very common to find eroded substrates clinically [36,54,59]. The importance of finding the best strategy and clinical plan to rehabilitate these cases has gained a significant weight. Gathering all the current evidence is key to clarify which is the best adhesive strategy when dealing with a clinical scenario of erosion.

Even though enamel is the first anatomic barrier exposed to acid challenge, and therefore subject to an erosion phenomenon, the majority of primary studies focused on dentin. In fact, the latter is commonly affected by erosion and evidence tells us that adhesion remains a challenge when held in dentin [21,32,44,56]. Moreover, most cavity preparations involve dentin, hence the need for evidence that can guide clinical in this substrate. When bond strength is the outcome, eroded enamel seemed not to be a challenge [37,50]. Eroded dentin, however, showed significant differences when compared to sound dentin [44,46]. Furthermore, authors that compared the performance of a self-etch and an etch-and-rinse adhesive [34], an etch-and-rinse with a universal adhesive [38] or a self-etch with an universal adhesive [39] all advocate that in the presence of erosion, the bonding performance may be compromised in dentin, even if some adhesives showed better results. Other studies also highlighted the fact that bond strength might be higher when enamel is eroded [46]. This is expected as the rougher surface that forms plays a favorable role in securing interlocking of the resin in enamel [13,37,54,56]. Giacomini et al. [50] even states that no additional treatment is required in eroded enamel, and pre-conditioning with 37% phosphoric acid may be enough to guarantee a successful and conservative bond. Despite this, Wang et al. [13] and Casas-Apayco et al. [54] argue that the changes in substrate such as loss of structure as well as disorganization present in eroded enamel should not be ignored. These alterations could lead to wear, mineral and consequently hardness deficiency, all factors that contribute to weakening of the substrate [13,37,54].

As for reestablishing mineral loss in dentin and trying to revert alterations caused by acid erosion, different pretreatments were tested. Some remineralizing agents like stannous-chloride and amine fluoride (SnCl2/AmF) were not persistent and did not increase the bond strength to eroded dentin nor did arginine-containing toothpastes [38,48]. In spite of these results, other types of remineralizing agents may lead to precipitation of calcium-fluoride-like deposits on the tooth surface, thus reducing the erosive mineral loss in dentin, as shown by Flury while experimenting with NaF and Sn/F solutions [55]. Krithi [34], on one hand, also demonstrated that sodium fluoride (NaF) showed improvement in bond strength results. On the other hand, the authors stressed the need for more studies regarding NovaMin [34], a type of bioglass composed of calcium sodium phosphor-silicate and usually indicated for dentin hypersensitivity [60]. Since remineralizing agents have a vast intervention field in operative dentistry and very few studies investigated these agents, further studies should be considered.

While remineralizing agents have not been consistent, deproteinizing agents demonstrate some significant results. Use of NaOCl previous to the application of the adhesive minimized the degradation of the latter, in long-term studies [39]. In fact, NaOCl pretreatment is capable of partially removing the organic superficial layer in eroded dentin and thinning the smear layer. This could be a solution to promote resin infiltration and thus bond strength to eroded dentin [41,43]. It is wise to underline that two of these three studies used NaOCl at 10% and the other one chose a concentration of only 5.2%, and therefore, a strict protocol is needed for more consistent results [39,41,43]. Another valid pretreatment is laser irradiation to reduce the superficial layer, affected by erosion, and modify the substrate. This prepares the surface for bonding without negatively affecting the substrate. Er,Cr:YSGG laser associated with a self-etch adhesive has shown higher bond strength results when compared to other surface pretreatments including diamond bur [57]. Maeda et al. [49] also demonstrated that Nd:YAG laser seems to have benefits in bond strength results, under erosive challenges. Finally, although conservative preparations and noninvasive treatments need to be respected, some treatments advocating the use of a fine-grift diamond bur led to better long-term results when dealing with eroded dentin. The rationale for this is the removal of the disorganized superficial layer, facilitating adhesion [43,58].

At baseline, bonding to sound dentin is already considered difficult and short-lasting. When dentin suffers erosion this difficulty increases even more, as explained above. As the literature shows, the establishment of a hybrid layer, composed of collagen, monomers and eventually debris (*smear layer)*, is key to establish an appropriate bond, being directly related to the chemical stability and longevity of the restoration [61,62]. Regarding eroded dentin, there is a dissolution of peri and intertubular minerals, resulting in the exposure of a thick superficial organic layer and, after restoration, tag formation of under 3 μm whereas for sound dentin, these values tend to be between 9 and 15 μm [25]. Collapsed demineralized fibrils and excess water content are also observable, leading to a deficient hybrid layer and impairing the penetration and *in situ* polymerization of the adhesive, ultimately affecting bond strength [17,47]. Since eroded dentin leads to an increase of water present in the matrix, nanoleakage becomes a bigger threat to hybrid layers [42].Consequently, this water is partially responsible for accelerated activation of endogenous proteases, the so-called matrix metallo-proteinases (MMPs), capable of hydrolyzing the organic matrix [63,64]. In fact, this lengthened contact between water and monomers found in the adhesive can ultimately lead to an accelerated hybrid layer degradation and failure of the restoration [58]. Events such as incomplete polymerization, resin plasticization or creation of water-rich channels seen under erosive conditions, are able to degrade chemical bonds within the polymer matrix and thus contribute to enzymatic degradation of the denuded collagen [65,66]. Accordingly, these studies highlighted the need to overcome the difficulties in infiltration of the adhesive to a thickened eroded organic layer as well as protecting a more vulnerable and challenged hybrid layer.

In order to answer and establish protocols that could protect the hybrid layer from early degradation, some authors led experiments and pretreatments with enzymatic inhibitors. As eroded dentin is more vulnerable to hybrid layer breakdown, it is important to stabilize the interface. In fact, MMPs can be activated by exposure to low pH and are able to progress dental erosion [63,67]. The successive pH demineralization-remineralization cycles are not sufficient to inhibit MMP activity, although their optimum pH to function is around neutral conditions [67,68]. Therefore, when dealing with erosive challenges, there is an accelerated activation of the proteases [43]. Chlorhexidine (CHX) has been often tested, since it was proven that it can inhibit proteolytic enzymes and reduce chances of collagen fiber degradation. Since eroded dentin produces a sensitive hybrid layer, testing inhibitors could be the subject of promising investigations. Even though it has been proven that it is only able to retard degradation and not fully inhibit it, 2% CHX was tested in a few studies included [36,40,43,47,51,53]. The results, however, were conflicting and do not support any recommendation [36,47,51]. Evidence has shown that even though CHX does not seem to have significant results on immediate bond strength, the substance might have beneficial effects over time in sound dentin and is considered a promising enzymatic inhibitor. However, CHX only remains effective when it is trapped in the dentin matrix, as the chemical bond is electrostatic and reversible [51]. The results also seem to be dependent on the adhesive used. In fact, CHX and 10-MDP may compete over the calcium present in apatite. That being said, CHX may reduce the immediate bond strength when used simultaneously with a universal or self-etch adhesive [36,47]. The type of adhesive and whether it is susceptible to inhibitors must be taken into account when using this type of pre-treatment. Moreover, some studies show that CHX causes a reaction when in presence of dentin, forming a precipitate that ultimately reduces the depth of dentin etched [36]. In the presence of eroded dentin, these results are even less predictable.

While CHX or benzalkonium chloride (BAC) showed that proteolytic inhibitors may not improve the durability of bond strength, other agents showed encouraging immediate bond strength results. In fact, cross-linking agents like proanthrocyanidins (PAA) and riboflavin (RFV) contributed to stabilize the collagen mesh, resulting in promotion of monomer infiltration, ultimately altering the bonding and nanomechanical properties of eroded dentin [32].

A variety of adhesive systems were taken into account in the studies included. The conclusions regarding this question were not consistent, since some studies did not compare different types of adhesives, and many contemporary adhesives were not featured. As mentioned before, bonding to enamel does not pose a problem and does not seem to be adhesive-dependent. Conversely, in dentin, most studies demonstrate that for several types of adhesives tested, bond strength in eroded dentin was compromised [34,42]. Nevertheless, the majority of authors suggest that the type of adhesive influences bond strength to eroded dentin, although the best adhesive strategy is still unknown with this amount of evidence [33,42,57]. As previously seen, one of the main obstacles is the lack of proper infiltration by the monomers in eroded dentin. Functional monomers such as 10-MDP have the ability to chemically interact with calcium present in hydroxyapatite. An intermediate layer where MDP molecules are bonded to the calcium in the surrounding solution and to another MDP molecule may be formed. This promotes long-term stability of the adhesive interface and higher short-term bond strength results. This so-called ‘nanolayering’ phenomenon is an advantage towards adhesives that only benefit from mechanical adhesion [69,70]. In eroded dentin, there is only a partial demineralization of the inorganic matrix and therefore, calcium is left to interact with 10-MDP. Thus, MDP-containing adhesives may be useful in this condition [42]. Furthermore, other materials such as self-adhesive flowable composites were tested. These presented very similar bond strength when compared to traditional composite bonded *via* an adhesive [35] and may be a promising strategy. Cruz et al. [59] and Lenzi et al. [56] compared adhesives to glass ionomer cements, although the aim is redundant as longevity associated to glass ionomers is limited. Despite this, a trend in certain types of adhesive choices was noted, especially in what concerns 3 M adhesives such as Adper Single Bond 2 or Scotchbond Universal. Further research should be conducted with other adhesives such as Clearfil Protect, Clearfil S3 and more studies featuring Optibond FL should be led, regarded as the gold standard of the etch-and-rinse category, and other formulations also used clinically.

Some questions still remain unanswered due to the lack of standardized protocols. All studies followed a specific erosive protocol but all showed differences. Most authors preferred citric acid [33,34,36–39,41,43,45,46,48,55,57,58], in most cases at a 1% concentration. Yet, not only the concentrations differed from one another but every study had a distinct management of the erosive cycling applied. While some applied citric acid to the samples four times a day for 5 min [46], others did the same but six times a day [58]. For instance, Cruz [59] concluded that bond strength was not affected when dealing with eroded dentin although when in 2015 [52] the same author changed erosive protocols, the results led to the belief that erosion had indeed compromised the quality of the bond. This reinforces the idea that the disparity of experimental conditions leads to conflicting results. Also, other authors operated with soft drinks such as Coca-Cola [13,32,35,42,44,51,53,54,56,59] or Sprite [49,52]. Some also used orange juice to simulate the effects of citric acid [47,50], while others chose hydrochloric acid. Differences in concentration of the acid, pH of the solutions used, demineralization times, total amount of days and type of sample all contributed to great disparities among the studies. A consensus for models of erosion in dental research, published in 2011 sets out recommendations [71]. The authors consider that citric acid is to be used as a model solution, preferred over commercial beverages due to its reproducibility and pH control. The duration of the challenge should be concordant with the aim of the study but should not last more than minutes, when it is a model for extrinsic erosion, and pH should be within an acceptable range found in real-life acidic challenges. The details of these protocols should be published in sufficient extent in these studies [71].

Eleven studies which used bovine teeth as substrate in alternative to human teeth were identified [13,36,40,43,45,48,49,51,53,55,58]. Past studies have documented that bonding to bovine teeth is comparably different than to human teeth, and results have to be cautiously interpreted [72,73]. Accordingly, it is always preferable to research with the most realistic conditions possible and in this case, some studies may lack that nature, eventually leading to suboptimal results. The duration of the acid challenge has to be adapted if a bovine substrate is used, as acid suscpetibility is different to that of human mineralized tissues [71].

Regarding bond strength tests, the preferred setup was the microtensile bond strength test [13,32,36,38–43,46–48,50,51,54,55,57,58]. Most authors chose this type of test mainly because it is highly reproducible, useful for material screening and has good clinical translation. Even though it lacks the ability to simulate intraoral conditions, since it does not take into account the C-factor, it is considered the gold standard in bond strength testing [74,75]. Additionally, Murase et al. [35] tested a new bond strength setup based on a new scratch and tensile test and future studies should take the opportunity to validate this method.

Gaps in the evidence surrounding adhesion studies in eroded substrates were identified. Novel remineralising composites, such as commercialized Activa Bioactive (Pulpdent) or experimental composites [76] are yet to be researched in the context of erosion. Sensitivity analysis of disparities in erosion protocols should be carried out, along with formulation of a standardized erosion protocol as guidance. A definitive need for in-depth research remains regarding adhesive options and clarifying the need for pretreatments is essential. Research in this topic should firstly focus on identifying methods to secure initial bonding to eroded dentin, rather than evaluating degradation. Mapping the preclinical evidence is important to guide future clinical studies by identifying which strategies are viable and which should not be further tested. Clear guidelines should be established in order to give clinicians better treatment options when dealing with erosive conditions. This can ultimately lead to better bond strength results which can translate to durable, successful restorations.

As a limitation, this scoping review deliberately focused in laboratory studies and did not include any type of clinical study, which could be interesting to compare with our present results in the future, in another scoping review or systematic review with a convergent research question. However, clinical studies evaluating adhesion in eroded susbtrates are scarce, and a scoping review with an aim of pre-clinical studies should precede clinical reviews. As one paper could not be retrieved, it also adds to the limitation of the present review, which would have contributed to the findings and qualitative synthesis.

## 5. Conclusion

A considerable amount of evidence was found regarding adhesion studies which measured bond strength in eroded substrates. Based on the evidence mapping performed in this study, the following conclusions can be drawn:

- Bond strength to eroded dentin is substantially reduced, compared to sound dentin, and this was a finding transversal to all studies. Enamel on the other hand, benefits from an erosion challenge during the adhesive procedure.
- Bond strength to eroded dentin is material-dependent, with best results seen in adhesive systems containing functional monomer 10-MDP.
- Surface pre-treatments such as bur preparation, laser irradiation (Er,Cr:YSGG; Nd:YAG) and NaOCl were able to improve bond strength to eroded dentin. Remineralization strategies and novel self-adhesive composites also showed promising results and warrant further research.
- MMP inhibitors in eroded dentin show conflicting results, with some authors supporting their use, and others reporting no effect at short and long-term or adverse effect in bond strength, specifically concerning 2% CHX.
- Standardization of laboratory studies is recommended. Studies should confirm the use of sound teeth prior to erosion protocols and properly randomize samples and allocate them to experimental groups. A non-eroded control group is highly recommended to serve as a baseline for data analysis. The erosion study model should specify whether it is extrinsic or intrinsic erosive simulation, and for extrinsic, citric acid is encouraged over soft drinks.
